# Thyroid Density in CT Imaging as a Potential Marker of Lung Involvement in COVID-19: A Retrospective Analysis

**DOI:** 10.7759/cureus.59699

**Published:** 2024-05-05

**Authors:** Suhasini Balasubramaniam, Aparna Suresh Kumar, Pravin Pandian, Pravin Kumar Raviganesh, Sowmiya Perumpallipatty Kumarasamy, Bharathi Priya Raju, Balaji Selvaraj, Amitesh Krishna Srinivasan, Sangeetha Balaji, Swaminathan Ramasubramanian

**Affiliations:** 1 Radiodiagnosis, Government Stanley Medical College and Hospital, Chennai, IND; 2 Internal Medicine, Government Medical College, Omandurar Government Estate, Chennai, IND; 3 Pharmacology, Government Medical College, Omandurar Government Estate, Chennai, IND; 4 Radiodiagnosis, Jawaharlal Institute of Postgraduate Medical Education and Research, Puducherry, IND; 5 Radiodiagnosis, Government Medical College, Omandurar Government Estate, Chennai, IND

**Keywords:** ct (computed tomography) imaging, radiological assessment, thyroid hormones, non-thyroidal illness syndrome, endocrine manifestations, sars-cov-2, thyroid density, lung involvement, thyroid dysfunction, covid-19

## Abstract

Background

The SARS-CoV-2 pandemic has underscored the multifaceted impact of the virus on human health, extending beyond the respiratory system to involve other organ systems, including the endocrine system. Emerging evidence suggests a notable interaction between COVID-19 and thyroid function, characterized by alterations in thyroid hormone levels and structural changes within the gland. This study aims to explore the association between thyroid density on CT imaging and lung involvement in patients with COVID-19, potentially offering new insights into the systemic effects of the virus.

Methodology

A retrospective cross-sectional analysis was conducted on 1,066 patients with COVID-19 who underwent chest CT scans without contrast at Government Medical College, Omandurar Government Estate, Chennai, which was designated as the COVID-19 care center from April to June 2021. Thyroid density and lung involvement were quantitatively assessed, and their correlation was analyzed using descriptive and inferential statistics, including the Kruskal-Wallis H test and Shapiro-Wilk test for normality.

Results

The study population predominantly exhibited normal thyroid density (749, 70.3%), followed by altered (212, 19.9%), nodular (104, 9.8%), and a single instance (0.1%) of absent thyroid density. Despite variability in lung involvement across different thyroid density categories, statistical analysis revealed no significant association between thyroid density and the extent of lung involvement in patients with COVID-19.

Conclusions

This study found no significant correlation between thyroid density and lung involvement in patients with COVID-19, suggesting that thyroid density on CT imaging may not serve as a reliable marker for lung involvement in this population. Further research is warranted to explore the complex interactions between COVID-19 and thyroid function, as well as the potential implications for patient management and prognosis.

## Introduction

The intricate relationship between SARS-CoV-2 infection and thyroid function is an interesting area of research, with evidence suggesting that thyroid abnormalities, including alterations in hormone levels and structural changes within the gland, are not uncommon sequelae of COVID-19. Previous findings have illuminated the prevalence of reduced free triiodothyronine (FT3) levels in these patients, potentially mirroring systemic inflammation and serving as a harbinger of clinical deterioration [1-4]. Moreover, the occurrence of non-thyroidal illness (NTI) syndrome or euthyroid sick syndrome (ESS) appears more frequently in severe cases of COVID-19, with a marked reduction in FT3 levels [1]. This underlines the capacity of the virus to both initiate new thyroid pathology and exacerbate existing conditions, as illustrated by Allam et al. through a case series that highlights increased thyroiditis, variations in thyroid volume, and intensified symptoms in thyrotoxic and hypothyroid patients [5].

The impact of COVID-19 on the thyroid extends to a wide array of disorders, from hyperthyroidism to hypothyroidism, indicating the virus's broad influence on thyroid health [5]. Furthermore, the immunological disturbances triggered by the infection, including cytokine storms and heightened immune responses, are posited to play a role in the etiology of destructive thyroiditis [6].

Survivors of COVID-19 may exhibit smaller thyroid gland volume, abnormal thyroid function, and even thyroid complications, which can impact vital organs and mortality rates [6-9]. Thyroid lesions incidentally discovered during cross-sectional imaging of the neck in patients undergoing examinations for unrelated reasons are commonly encountered and frequently missed. Additionally, the incidence of thyroid incidentalomas, nodules, and lesions is higher in patients with COVID-19 compared to the general population [9-11]. This oversight not only poses clinical challenges but also imposes a notable economic burden due to the need for further evaluation and management [11]. The fluctuations in thyroid hormone metabolism, particularly the reduction in free thyroxine (FT4) and FT3 levels in severely ill patients have prognostic significance and may act as biomarkers for disease severity [12]. The concentration of thyroglobulin (TG) tended to decrease over time, particularly in patients treated with glucocorticoids (GCs). TG levels did not differ significantly between patients with normal and abnormal TFTs [13].

Early detection of these abnormalities, facilitated by thyroid gland assessment in CT scans, is crucial for timely intervention and management [9,11]. This study seeks to extend these findings by investigating the potential correlation between varying thyroid densities on CT scans and the course of COVID-19, to enrich our understanding of the virus' impact on thyroid pathology. This research may uncover novel prognostic tools and contribute to more refined management strategies for patients with COVID-19, offering a clearer picture of the disease's systemic reach and its implications for thyroid function.

## Materials and methods

Study setting and design

This research was a retrospective cross-sectional analysis carried out within a hospital environment, concentrating on the evaluation of CT scans of the chest. The study received approval from the Institutional Ethics Committee, cited as IEC No. 104/IEC/GOMC/2023, and was conducted in strict adherence to the ethical guidelines recommended by the institutional research board, aligning with the ethical standards of the 1964 Declaration of Helsinki and its later amendments. The Department of Radiodiagnosis at the Government Medical College, Omandurar Government Estate, Chennai, undertook the research. This college is linked with The Tamil Nadu Dr. M.G.R. Medical University and was recognized as a COVID-19 care center during the start of the pandemic.

Study period

The research spanned from April 2021 through June 2021, providing a detailed period for in-depth data examination.

Participants

Individuals aged 18 years and above were included as study participants.

Inclusion criteria

To be considered for this study, patients needed to be at least 18 years old at the time of admission to the medical facility. Eligibility was also determined by the presentation of clinical symptoms associated with COVID-19, such as fever, cough, difficulty breathing, tiredness, muscle or body aches, headaches, loss of taste or smell, sore throat, congestion, nausea, vomiting, and diarrhea, along with a verified positive COVID-19 test result [14].

Exclusion criteria

Subjects under the age of 18, those not exhibiting clinical symptoms of COVID-19, or lacking a positive RT-PCR test confirming COVID-19 were excluded.

Sample size

The study involved 1066 patients who fit the inclusion criteria, identified through convenience sampling.

CT chest imaging protocol

CT scans of the chest without contrast were conducted on a 16-slice multidetector CT scanner (Aquilion Lightning model TSX-035A, Toshiba America Medical Systems, Tustin), with patients lying supine and holding their breath in inspiration. The images were analyzed using Vitrea software version 6.5.99 (Vital Images, Inc., Otawara, Japan). The imaging parameters included a window width of 1,000 to 2,000 Hounsfield units (HU) and a window level of -700 to -500 HU, ensuring coverage of the entire lungs from the root of the neck to costophrenic angles. Two experienced radiologists, with a combined experience of 15 years, initially reviewed the images, which were then reassessed in conjunction with residents and interns. A double-blind approach was used to assign images for review. Any interpretative differences were settled by a consensus meeting involving a senior radiologist with 20 years of expertise.

Quantification of pulmonary involvement and thyroid density

Lung Involvement (LI) was assessed in 20 sections, with scores based on opacification: two for over half involvement and one for less than half. The total maximum score was 40, each unit representing 2.5% of affected lung parenchyma [15]. Based on the score, LI was represented as 0 to 1 corresponding to 0 to 100 percentage points. Thyroid was categorized into four distinct types: normal, altered, nodular, or absent. An altered thyroid was identified by a density of less than 70 HU, while the presence of hypodense nodules was indicative of a nodular thyroid [16].

Data management

Data from CT images and patient records were meticulously recorded in a Microsoft Excel spreadsheet (Microsoft® Corp., Redmond), adhering to rigorous confidentiality and privacy measures.

Statistical analysis

To examine the link between thyroid density and LI, we applied descriptive and inferential statistics. Descriptive measures included mean, standard deviation, median, interquartile range (IQR), and range for LI, alongside frequency counts for thyroid density categories (absent, altered, nodular, normal). The Shapiro-Wilk test assessed the normality of LI distributions. Based on normality results, the Kruskal-Wallis H test was used for comparing LI across thyroid categories for non-normally distributed data, with post hoc Dunn's test and Bonferroni correction for identifying specific group differences. Visualization histograms, boxplots, and violin plots aided in displaying data distributions and group comparisons. Analyses were performed using Python 3.9 (Python Software Foundation, Wilmington), employing pandas for data handling, scipy for statistical testing, and matplotlib and seaborn for visualization.

Ethical considerations

The research conducted adhered strictly to the ethical principles outlined by our institution, with a primary focus on safeguarding the confidentiality and anonymity of patient information. To ensure anonymity, patient identification particulars were removed utilizing the Picture Archiving and Communications System (PACS) software (GE Healthcare, Chicago). Following this, distinctive random numerical identifiers were allocated to each image and clinical data entry to enable association while preserving anonymity. The data was securely stored within a password-protected folder on the Department of Radiodiagnosis computer system. All procedures involving human subjects were performed in accordance with the ethical guidelines established by the institutional and/or national research committee.

## Results

**Figure 1:**
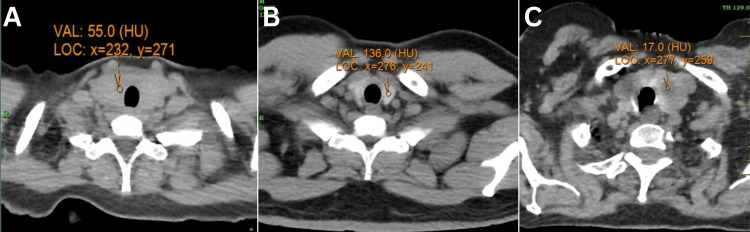
CT thyroid images. (A) Enlarged thyroid gland with diffuse hypodensity, 55 HU (<70); (B) normal thyroid gland, 136 HU (>70); (C) multiple hypodense nodules are seen in both lobes of the thyroid gland. The left lobe is enlarged due to the presence of the nodules.

This study explored the association between thyroid density and lung involvement among patients with COVID-19. The dataset comprised 1,066 observations, detailing the categorization of thyroid density into four distinct groups: *Absent*, *Altered*, *Nodular*, and *Normal*, along with measurement of lung involvement for each subject. Examples illustrating *Altered,* *Nodular*, and *Normal* categories are shown in Figure 1.

**Figure 2:**
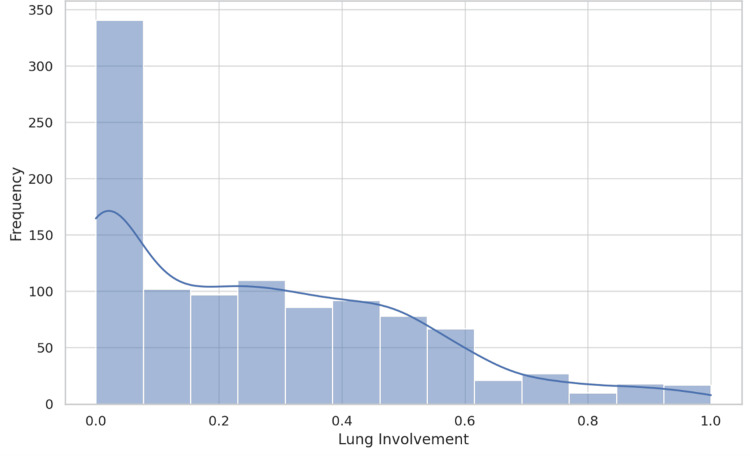
Histogram of lung involvement. This histogram provides a visual representation of the distribution of lung involvement across all participants in the study. The x-axis represents the extent of lung involvement, while the y-axis indicates the frequency of occurrences within the dataset. The shape of the distribution, as shown by the histogram, suggests a skew toward lower values of lung involvement, with a tail extending toward higher values.

**Figure 3:**
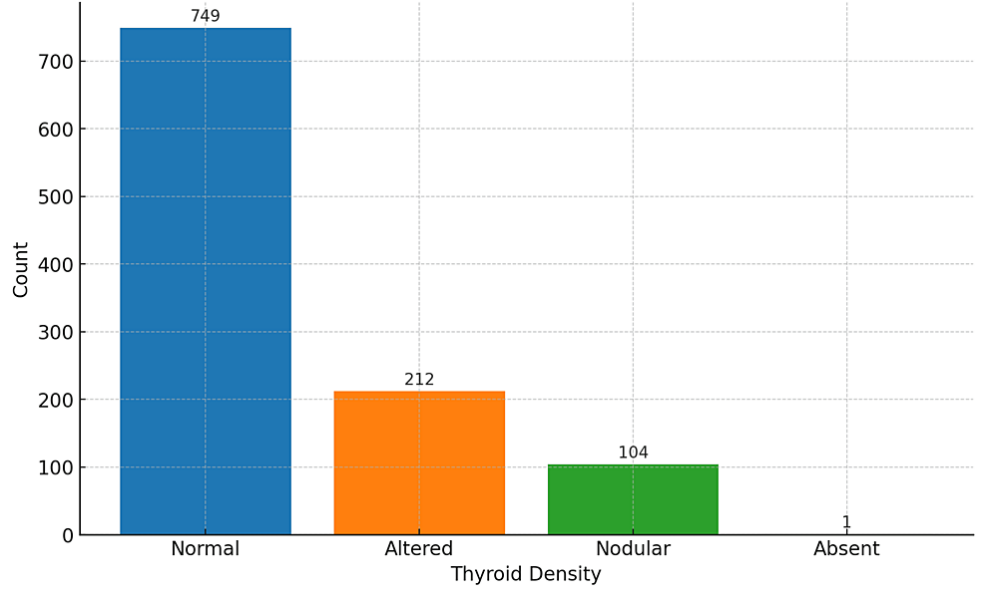
Count plot of thyroid density categories. The count plot displays the frequency of each thyroid density category within the study population. The x-axis categorizes the thyroid density into *Normal*, *Altered*, *Nodular*, and *Absent*, while the y-axis counts the number of occurrences within each category.

The descriptive statistics for lung involvement revealed a mean value of 0.270, with a standard deviation of 0.246. The range of lung involvement spanned from 0.000 to 1.000, indicating a wide variability among the subjects. The distribution of lung involvement, as depicted in Figure 2, showed a skew toward lower values with a presence of higher measurements across the dataset. Regarding the distribution of thyroid density categories, *Normal* was the most prevalent classification, accounting for approximately 70.3% of the observations, followed by *Altered* (19.9%), *Nodular* (9.8%), and a single instance of *Absent* thyroid density (Table 1). The count plot in Figure 3 illustrates the distribution of these categories within the dataset.

**Table 1:** Distribution of thyroid density categories.

Thyroid density	Count	Percentage
Normal	749	70.3%
Altered	212	19.9%
Nodular	104	9.8%
Absent	1	0.1%

**Figure 4:**
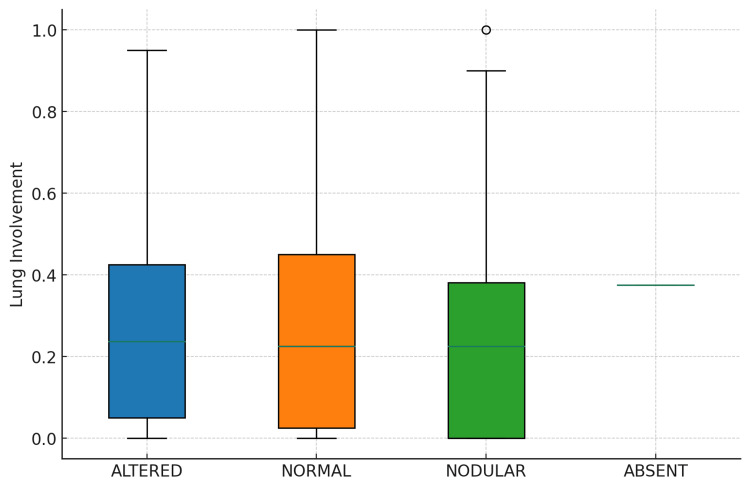
Boxplot of lung involvement by thyroid density category. This boxplot compares the distribution of lung involvement across the different thyroid density categories. Each box represents the interquartile range (IQR) of lung involvement for a category, with the horizontal line inside the box marking the median. Whiskers extend from the box to show the range of the data, excluding outliers, which are represented as individual points.

**Figure 5:**
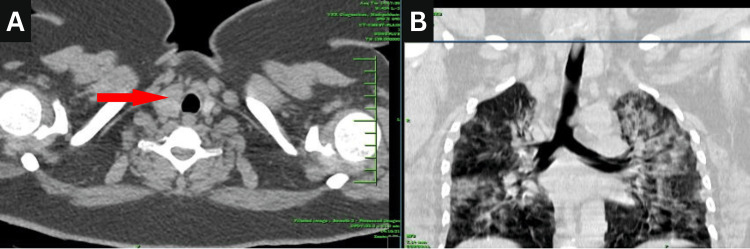
CT chest and thyroid of a patient with COVID-19. (A) Nodules in the right lobe of the thyroid gland, 58 HU; (B) patchy areas of the parenchymal crazy paving pattern of consolidation (60% lung involvement) in the bilateral lung parenchyma in a patient with RT-PCR swab positive for COVID-19. RT-PCR, reverse transcription polymerase chain reaction

The relationship between thyroid density and lung involvement was further examined through a boxplot analysis, revealing variability in lung involvement across the different thyroid density categories (Figure 4). *Nodular* thyroid density exhibited a wider interquartile range and higher median lung involvement than *Normal* and *Altered*, suggesting potential differences in lung involvement across categories. The Shapiro-Wilk test for normality returned a *P*-value significantly less than 0.05, indicating that the lung involvement data did not follow a normal distribution. Consequently, the Kruskal-Wallis test was employed to assess differences in lung involvement across thyroid density categories. The test yielded a statistic of 1.0134 with a *P*-value of 0.7980, suggesting no significant differences in lung involvement across the thyroid density categories. Figure 5 shows a CT image of the chest and thyroid of a patient.

Table 2 presents the basic statistics of lung involvement for each thyroid density category. For the *Altered* category, the mean lung involvement was 0.279, with a standard deviation of 0.258, ranging from 0.000 to 0.950. The *Nodular* category showed a mean of 0.254, with a standard deviation of 0.241 and a range extending to 1.000. The *Normal* category had a mean lung involvement of 0.269, a standard deviation of 0.244, and the same maximum value of 1.000 as the *Nodular* group. These findings highlight the variance within and between thyroid density categories regarding lung involvement.

**Table 2:** Descriptive statistics of lung involvement by thyroid density.

Thyroid density	Mean	Standard deviation	Min	25%	Median	75%	Max
Altered	0.279	0.258	0.000	0.050	0.2375	0.425	0.950
Nodular	0.254	0.241	0.000	0.000	0.2250	0.381	1.000
Normal	0.269	0.244	0.000	0.025	0.2250	0.450	1.000
Absent	0.375	-	0.375	0.375	0.3750	0.375	0.375

**Figure 6:**
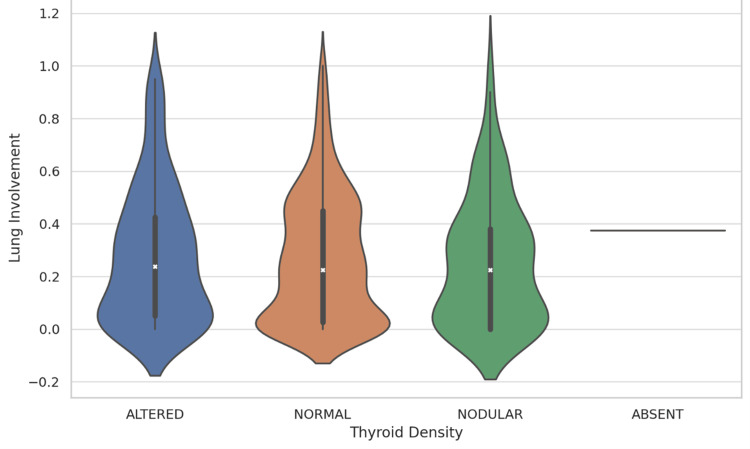
Violin plot of lung involvement by thyroid density. Each *violin* shows the density of data points at different lung involvement levels, with the thickness of the shape indicating the density. Internal markers denote the median and interquartile ranges.

The violin plot, depicted in Figure 6, illustrates the presence of variability and skewness in lung involvement distributions within each category. Specifically, it shows the density of data points at different levels of lung involvement, alongside the interquartile range and median, represented within the shape of each violin. The *Nodular* category displayed a broader distribution, indicating a wider range of lung involvement values. In contrast, the *Normal* and *Altered* categories showed narrower distributions, suggesting less variability in lung involvement among these groups. The singular observation in the *Absent* category is also represented, providing a complete overview of the dataset.

The insights gained from the violin plot underscore the variability in lung involvement across thyroid density categories. While the Kruskal-Wallis test indicated no statistically significant differences in lung involvement across categories, the visual representation provided by the violin plot reveals the nuances in the data distribution that might not be captured by summary statistics or traditional hypothesis testing alone.

## Discussion

The COVID-19 pandemic has illuminated various systemic effects beyond the initial respiratory implications, including the endocrine system. Our study found that most individuals had normal thyroid density (749, 70.3%), followed by altered (212, 19.9%), nodular (104, 9.8%), and one case (0.1%) of absent thyroid density. While lung involvement varied across thyroid density categories, statistical analysis showed no significant association between thyroid density and the extent of lung involvement in patients with COVID-19.

Thyroid density alterations in patients with COVID-19 have been a focal point in discerning the virus's systemic impact. Akkoyunlu et al.'s findings of diminished thyroid densities in patients with COVID-19, correspond with heightened mortality rates. Our study extends this narrative by correlating these thyroid density changes with the severity of lung involvement, underscoring the potential of thyroid imaging parameters as prognostic tools in assessing COVID-19 severity. This correlation invites further investigation into whether these imaging findings can serve as a predictive measure for patient outcomes [7]. Additionally, the reduction in thyroid gland volume reported by Urhan et al. in survivors of COVID-19 postulates a transient or acute effect of SARS-CoV-2 on thyroid function, reflected by lower fT3 levels during infection [6]. While these findings did not persist long-term, the acute phase alterations provide a window into the immediate impact of the virus on thyroid function, which could be relevant for acute management of patients with COVID-19.

The prevalence of incidental thyroid nodules in patients with COVID-19, as reported by Abdelrahman et al. and Jantarapootirat et al., raises compelling considerations for routine imaging practices during the pandemic [9,10]. These studies collectively suggest an increased prevalence of thyroid nodules in patients with COVID-19 compared to the general population, which may imply a direct viral effect or an indirect consequence of systemic inflammation. Our study did not delve into nodule prevalence per se; however, the observed variations in thyroid density may be tangentially related to nodule formation, raising questions about the potential long-term implications of these incidental findings. The presence of incidental thyroid nodules identified through imaging raises a pivotal question regarding their clinical significance. While many nodules are benign and may not bear immediate clinical consequences, the heightened prevalence observed in patients with COVID-19 suggests an intersection between viral infection and thyroid pathology that could facilitate early detection of thyroid malignancies, thereby influencing overall patient management [9].

The broader implications of COVID-19 on the renin-angiotensin system (RAS) and potential therapeutic interventions, such as the use of RAS inhibitors, including vitamin D and sartans, present an intriguing avenue for mitigating the extrapulmonary effects of the virus. Given the role of the RAS in thyroid pathology, particularly through the ACE2 receptor and AT1R receptor interactions, RAS inhibitors emerge as potential therapeutic agents. Their role in mitigating the adverse effects of the viral Spike protein on the thyroid gland posits a targeted approach to managing COVID-19-induced thyroid dysfunction [17]. The systemic nature of COVID-19 is further emphasized through the observation of overt thyroid disorders in critically ill patients, with thyrotoxicosis being a notable condition. The association of larger nodules with disease severity, albeit not conclusively linked, warrants attention to the thyroid axis in the acute management of patients with COVID-19 [12]. Such associations suggest that thyroid dysfunction may not only be a comorbid condition but potentially a contributing factor to the pathogenesis of severe COVID-19. Clinical presentations of thyroid dysfunction in COVID-19, such as weight gain, psychomotor retardation, and sensations of cold, are indicative of a hypothyroid state, often aligning with low circulating thyroid hormone levels. The emergence of chronic autoimmune thyroid conditions post-infection or post-vaccination further illustrates the enduring influence of COVID-19 on the thyroid axis [17].

Zhang et al.'s application of a Mendelian randomization approach introduces a genetic perspective by suggesting a protective role of higher thyroid hormone levels against severe COVID-19 manifestations [18]. This genetic predisposition underscores the multifactorial nature of COVID-19 outcomes and the potential interplay between genetic factors and thyroid function. Although our study did not directly address genetic factors, the integration of genetic predispositions into our understanding of thyroid function in patients with COVID-19 could significantly enhance the predictive accuracy of clinical outcomes.

In analyzing these findings, it becomes apparent that the relationship between COVID-19 and thyroid function is dynamic. The convergence of imaging findings, genetic predispositions, and potential therapeutic interventions underscores the complexity of COVID-19 as a systemic illness, meriting a multidisciplinary approach to research and clinical practice.

## Conclusions

While the exploratory analysis suggested potential differences in lung involvement across thyroid density categories, statistical testing revealed no significant association between thyroid density and lung involvement within this dataset. The absence of significant differences indicates that, within the parameters of this study, thyroid density does not predict the extent of lung involvement. This conclusion is supported by the comprehensive statistical analysis, including both descriptive and inferential statistics, which underscores the complex nature of thyroid-related conditions and their potential impact on lung health.
